# VitalCSI: Contactless Respiratory Rate Estimation Using Consumer-Grade Wi-Fi Channel State Information

**DOI:** 10.3390/s26010225

**Published:** 2025-12-29

**Authors:** Tom Michaelis, João Jorge, Nivedita Bijlani, Mauricio Villarroel

**Affiliations:** 1 Institute of Biomedical Engineering, Department of Engineering Science, University of Oxford, Oxford OX3 7DQ, UK; tom.michaelis.tm@gmail.com (T.M.); joao.gm.jorge@gmail.com (J.J.); 2 The Podium Institute for Sports Medicine and Technology, University of Oxford, Oxford OX3 7DQ, UK; nivedita.bijlani@eng.ox.ac.uk

**Keywords:** respiratory rate (RR) monitoring, Wi-Fi, channel state information (CSI), continuous monitoring

## Abstract

Continuous respiratory rate (RR) monitoring can improve the detection of clinical events, such as pulmonary infections, cardiac arrests, and sleep apnoea. Wi-Fi-based systems offer a low-cost, contactless alternative to radar and video. However, existing studies are limited to narrow respiratory ranges and small-scale validation. We present *VitalCSI*, a vital sign monitoring system using off-the-shelf, low-power Wi-Fi hardware. We recorded 15 healthy university athlete volunteers and developed RR estimation algorithms benchmarked against nasal airflow sensors. *VitalCSI* uses a consumer Wi-Fi access point and a Raspberry Pi computer to capture channel state information (CSI). We estimated the RR from CSI via principal component analysis (PCA), spectral peak detection, and breath (counting in 30 s windows), which were then fused by a multidimensional Kalman filter. *VitalCSI* showed strong agreement with airflow references ($r^{2}=0.93$, MAE = 1.20 brpm), tracking RR across 6–33 brpm and outperforming prior Wi-Fi studies. *VitalCSI* demonstrates the feasibility of RR monitoring with a single-antenna, single-board microcomputer as the Wi-Fi transmitter. It is the first validated system for continuous, contactless RR monitoring using consumer-grade Wi-Fi over an extended respiratory range, paving the way for use in both home and sports monitoring contexts.

## 1. Introduction

Respiratory rate (RR) is a key vital sign for predicting clinical deterioration, including cardiac arrest [1], respiratory failure [2], ICU admission [3], and in-hospital mortality [4]. It also plays an important role in diagnosing and tracking conditions such as pulmonary infections [2] and obstructive sleep apnoea [5]. Continuous RR monitoring enables timely clinical intervention and improves patient outcomes [6]; yet, RR is rarely recorded outside clinical settings.

Manual breath counting remains the standard in many healthcare environments. This method involves a medical practitioner counting chest wall movements, typically over 60 s, though these are often shortened to 15 s and scaled up [7]. It is error prone, time consuming, and introduces the “white coat” effect as patient awareness of counting alters natural breathing. Despite these limitations, it remains the default method in non-critical care. However, its reliance on trained personnel renders it impractical for continuous or remote monitoring. Contact-based automated methods include flow-based sensors (e.g., nasal airflow) and motion-based systems, like impedance pneumography and inductance plethysmography. The former typically requires a face mask, which can cause discomfort, inhibit speech, and disrupt breathing [8]. The latter uses electrodes or chest bands to detect mechanical chest expansion, but these can irritate the skin, slip during movement, and are unsuitable for long-term use [9,10].

These limitations have driven the development of non-contact RR monitoring technologies, primarily using radar [11] and video [12]. Camera-based approaches track subtle chest movements but require line-of-sight, consistent lighting, and can raise privacy concerns. Radar-based systems estimate the RR from skin displacement by detecting changes in the reflected electromagnetic (EM) waves [13,14], but they rely on specialised hardware, making them difficult to scale into home environments. The COVID-19 pandemic also underscored the importance of non-contact vital sign monitoring and highlighted a pressing, unmet need for a low-cost, non-intrusive, and scalable RR estimation system that utilises commercially available hardware.

Wi-Fi-based RR monitoring takes advantage of the ubiquity of consumer-level Wi-Fi equipment. Wi-Fi devices harness short-range radio frequencies, transmitting in the 2.4-to-5 GHz band. There exists a reflection and absorption effect between the human body and Wi-Fi electromagnetic radiation. At 5 GHz, the absorption cross-section of the human body—a measure of the effective area that absorbs incident RF waves—is approximately 0.2 m^2^ [13]. To optimise data transmission, Wi-Fi devices continuously estimate the signal strength between the transmitter and receiver. Thus, human respiration, which induces subtle perturbations in the Wi-Fi signal, can be analysed to infer RR.

Short-range radio communication is highly susceptible to multipath effects, where signals travelling along different paths arrive with varying delays and cause interference at the receiver. To address this, modern Wi-Fi systems employ orthogonal frequency division multiplexing (OFDM), which splits data across multiple orthogonal subcarriers and uses quadrature amplitude modulation (QAM) to support higher transmission rates [15]. The number of subcarriers scales with the bandwidth, from 64 at 20 MHz to 256 at 80 MHz. Further improvements arise from Multiple-Input Multiple-Output (MIMO) systems, which were introduced with the 802.11n standard [16] and exploit spatial multiplexing to transmit independent data streams along separate paths. When combined with beamforming, MIMO can dynamically steer signals toward favourable spatial channels, improving throughput and link robustness [15]. Two primary research approaches have emerged in Wi-Fi-based vital sign monitoring: techniques that measure the Received Signal Strength Indicator (RSSI) and those that measure the channel state information (CSI) of a Wi-Fi data link.

The RSSI is a relative measurement of the strength of a received signal measured at the receiver’s antenna. The RSSI is determined by the transmission power, the distance between the transmitter and the receiver, and the environment. The RSSI is often made available to users in many IEEE 802.11 devices and is used to assess the quality of available Wi-Fi connections. Established network analyser tools, such as Wireshark [17], can be used to extract RSSI data. Since the human body is an absorber of RF signals, the RSSI of an RF link is reduced by the presence of a human between the transmitter and receiver. The periodic movements during each breathing cycle cause subtle changes in the human thorax and surrounding air volume, which in turn modulate the propagation path and absorption characteristics of the Wi-Fi signal, leading to measurable fluctuations in the RSSI. Analysis of the RSSI can, therefore, be utilised for the detection and localisation of subjects, as well as the estimation of the RR [18,19,20].

RSSI-based methods for detecting physiological vital signs are limited. The conversion of signal power into low-resolution RSSI values reduces the granularity needed for accurate sensing, introducing a quantisation error [21]. As a result, the signal-to-noise ratio (SNR) of the respiratory signal extracted from the Wi-Fi data is reduced. RSSI methods also require the subject to be placed in the line-of-sight between the transmitter and receiver to ensure breathing has a large effect on the RSSI waveform. RR error doubles when the subject moves 0.13 m away from the line-of-sight, rapidly increasing when the separation of the receiver and transmitter is increased beyond 2 m. The multipath effect of MIMO Wi-Fi propagation means that there are spatial streams that reflect off walls and objects and are not transmitted through the subject. This further degrades the SNR of the breathing component in the RSSI signal.

Channel State Information (CSI) offers fine-grained insights into the wireless channel by capturing the amplitude and phase characteristics at the subcarrier level. This enables the extraction of comprehensive information, such as the path loss, delay spread, Doppler shifts, and multipath propagation effects between the transmitter and receiver [22]. CSI acquisition is underpinned by MIMO systems. They employ multiple transmit and receive antennas to improve data throughput, link reliability, and spatial resolution. By exploiting spatial diversity, MIMO enables the simultaneous transmission of independent data streams, thereby increasing sensitivity to subtle environmental changes, such as human respiration. Spatial multiplexing and beamforming are two mainstay techniques in MIMO systems that rely on accurate channel state estimates to improve signal transmission.

CSI-based vital-sign estimation can be categorised into techniques that analyse the phase of the received CSI signal and those that use the magnitude. Phase-based methods are limited in their utility as the phase of the CSI data, when using commodity Wi-Fi chipsets, has been shown to often be corrupted. This is mostly caused by packet detection delay at the receiver, device sampling frequency offsets, and random time-invariant phase offset [23]. When no methods are employed to correct for this distortion, the phase component is primarily composed of white noise [24]. The majority of modern research has focused on utilising the magnitude of the received CSI signal since magnitude measurements are more stable and retain usable motion-induced variations, making them more practical for vital-sign sensing on commodity Wi-Fi devices. In magnitude-based methods, each CSI entry is typically represented by 16–20 bits [25], ensuring low quantisation error. CSI is computed for each subcarrier across all transmit–receive antenna pairs, offering robustness to multipath effects. As a result, CSI-based methods are generally preferred over the RSSI for respiratory monitoring.

Wi-Fi systems offer key advantages. Unlike cameras, they do not require line-of-sight or ambient lighting. Unlike radar, they can use the commodity hardware already deployed in most homes. Over two-thirds of the global population now has access to Wi-Fi [26], including 94% of adults in the UK who have home Wi-Fi access [27]. Wi-Fi-based monitoring presents fewer privacy concerns as it does not capture visual or audio data, making it especially suited for use in bedrooms or other sensitive settings. A Wi-Fi-based system would require no operator, reducing infection risk and enabling autonomous patient monitoring in private settings without additional infrastructure.

Despite its potential, research in this space remains limited. Most Wi-Fi-based RR studies are proof-of-concept experiments using out-of-date Wi-Fi standards or specialised hardware. Extracting CSI data requires specialised hardware and software, as most consumer devices do not expose the CSI data by default. Currently, there is no standardised tool for extracting CSI data. The algorithms proposed by the current literature are susceptible to noise or motion artefacts, and they do not use all of the data available from the multiple subcarriers present in a Wi-Fi communication data link. Existing studies typically involve small cohorts (around six healthy volunteers) and only cover resting RR values (12-to-20 brpm) [28], neglecting the wider range of respiratory variability seen in real-world conditions. To realise the potential of Wi-Fi for RR monitoring, there is a clear need for systems built on modern hardware that are validated on larger populations and designed to function across broader respiratory ranges. Such systems would support the development of reliable, scalable algorithms suitable for real-world home use.

In this work, we make the following contributions:

1. The development of *VitalCSI*, a prototype system for collecting CSI using off-the-shelf wireless hardware that is compliant with modern Wi-Fi standards.
2. The release of open-source tools to support future research into the use of CSI for non-contact physiological monitoring.
3. The development of RR estimation algorithms, with high agreement with reference methods, validating the feasibility of Wi-Fi-based respiratory monitoring in a controlled setting.

Although the present study focuses on a controlled, seated breathing protocol, the participant cohort—of whom 73% had experience in competitive sports—provides an early indication of the work’s potential relevance to athletic monitoring, where unobtrusive assessments of respiratory dynamics could offer value in training and recovery contexts.

## 2. Related Work

### 2.1. Phase-Based Vital-Sign Estimation Algorithms

Zhang et al. proposed *BreathTrack* [29], a phase-based vital sign estimation system that detects small movements caused by breathing. It uses a reference antenna on the transmitter and a coaxial cable between the transmitter and receiver to correct for the time-varying phase components. The cable provides a path that is unaffected by changes in the environment. All phase variation is assumed to be induced by the wireless hardware. The measured phase distortion is then used to correct the phase delay between the remaining receiver–transmitter pairs. *BreathTrack* also proposes an angle of arrival and time-of-flight detection algorithm to eliminate multipath effects so that only the phase of the dominant path is used. It utilises a Hampel filter and high-pass filter to process the data. The Hampel filter aims to remove outliers by applying a sliding median filter over a window of time series. The RR is then estimated by taking the peak of the signal in the frequency domain. The use of a cable to correct the CSI phase incurs additional hardware cost and hinders the usability of the proposed Wi-Fi system as a practical vital sign monitoring solution.

### 2.2. Magnitude-Based Vital-Sign Estimation Algorithms

Wi-Covid [30] processes the CSI signal by using a Hampel filter and bandpass filter, between 0.2 Hz and 0.4 Hz, to estimate the RR between 12 and 24 brpm. Principal component analysis (PCA) is used to extract the RR component. A wavelet transform is then applied to generate a spectrogram from the PCA components, whose peaks are used to estimate the RR. Wi-Covid provides no description of the dataset used or any quantitative analysis of results, making evaluation and comparison of the proposed methods difficult. No methodology is provided as to how the respiratory component is selected from the set of all PCA components.

Liu et al. [31] proposed a method to track the breathing rate and heart rate during sleep. The proposed system uses a Hampel filter and moving average filter on each CSI subcarrier to remove high-frequency noise. The subcarriers with the highest variance in each sliding window, equivalent to the highest signal power, are selected for vital sign estimation. A peak detection algorithm is then applied to each selected subcarrier. The algorithm detects all peaks in a windowed signal, and the peaks that are not local maxima (or yield peak-to-peak intervals outside the physiological range of human breathing), are discarded. The algorithm computes the RR estimates by using a weighted mean based on the variance of each subcarrier for each window.

Kontou et al. [32] presented a low-cost RR monitoring system that captures the CSI amplitude from a commercial 5 GHz Wi-Fi router using a Raspberry Pi 4 with Nexmon firmware. A traffic-generating laptop ensures 100 Hz packet capture. CSI is calibrated (RSSI-based scaling), denoised via Hampel and smoothing filters, and extracted using PCA to yield a single respiration waveform. A two-layer artificial neural network is entirely trained on synthetic cosine waves spanning 0.1–1 Hz (≈6–60 brpm) in 0.005 Hz steps. The output was validated by comparison to FFT peak detection, giving a mean frequency error of ≈4.7%. However, all measurements were collected from one subject and only 20 short recordings. The ANN was trained only on simple sinusoidal signals in the 0.1–1 Hz range, which may not capture natural irregularities or higher respiratory rates.

WiResP [33] uses commercial 802.11ac hardware (5.8 GHz, 40 MHz, 30 Hz sampling, and two Tx × 1 Rx antennas). The system first denoises and normalises CSI amplitude, computes an auto-correlation function (ACF) for each subcarrier, and it then combines the strongest subcarriers by the breathing-to-noise ratio (BNR). The concatenated ACF spectra are then treated as images. A histogram-equalisation spectrum-enhancement step sharpens breathing traces, and an Otsu-thresholded continuity checker rejects false positives before estimating the RR from the lag of the dominant ACF peak. Ground truth is provided by a SleepBreathe nasal mask, allowing precise overnight validation across 35 full-night datasets (280 h) under line-of-sight and non-line-of-sight conditions. WiResP achieved ≈92% detection and <3% false-alarm rates, with median RR errors of ≈0.8–0.9 brpm, even when the access point was in a different room. It outperformed contemporary systems (SMARS, FarSense, etc.) by >25% in detection coverage. Key limitations are the single-subject focus and computational cost arising from frequent subcarrier BNR selection and image-based enhancement.

Burimas et al. [34] used two ESP32 microcontrollers operating at 2.4 GHz with a 22 MHz channel width, one as the Wi-Fi access point and one as the station, to capture the CSI amplitude while volunteers slept. The raw CSI was preprocessed through a multi-stage pipeline of Hampel outlier filtering, Gaussian smoothing, linear interpolation to 60 Hz, and Butterworth low-pass filtering to isolate breathing rhythms. Residual motion artefacts were detected with a *z*-score-based anomaly detector, and the final RR was estimated via a local peak counting method scaled to brpm. The ground-truth RR was provided by a Vernier Respiration Monitor Belt (4 Hz sampling). Eight adults of varying BMI each contributed 5 min of data, breathing at normal (12–16 brpm), slow (<12 brpm), and fast (>16 brpm) rates. Under optimal filter settings, the system achieved an overall mean absolute deviation (MAD) of 2.60 brpm, with the best performance for normal breathing (1.38 brpm) but higher error for slow (≈3 brpm) and fast breathing (≈1.8–3 brpm). This ESP32 approach stands out for ultra-low cost and simplicity but sacrifices physiological range and recording duration.

### 2.3. Phase and Magnitude-Based RR Estimation

Wi-Breath [35] captures amplitude and inter-antenna phase-difference signals at 5 GHz using an Intel 5300 NIC (3 Rx) and Mi Router 3 (2 Tx). The system derives the instantaneous RR from chest movements in the physiological range of 12–30 brpm (≈0.2–0.5 Hz) using peak-to-peak intervals in a 5 s sliding window. Eighteen candidate signal-quality features (e.g., SNR, amplitude/phase range, standard deviation, entropy, and peak-based respiration statistics) are initially extracted from each 30-subcarrier CSI stream. A backward elimination algorithm iteratively removes the least informative features, yielding a final 7-feature set for classification. These, along with reference respiration-band labels, are input into a linear-kernel SVM, and they are trained with repeated 10-fold cross-validation. This supervised classifier achieves about 91% accuracy and 17% lower error than amplitude-only baselines under optimal conditions (50 Hz sampling and 2.5 m Tx–Rx separation). This study involved only five healthy volunteers. The SVM model is likely to overfit.

WiRM [36] is a two-stage CSI-based algorithm that extracts the respiratory waveform and then estimates the RR. It uses Intel 5300-type Wi-Fi CSI (2 Tx × 2 Rx MIMO, 9.9 Hz sampling, and 114 subcarriers) and conjugate multiplication across receiving antennas to suppress the common-phase noise. In the RR estimation stage, conjugate-multiplied CSI magnitudes and phases undergo autocorrelation and BNR combining. This is followed by a zoom-FFT spectrogram and adaptive multi-trace carving (AMTC) that tracks frequency traces with a Gaussian transition model. This reduces the RR root mean squared error by ≈38% and yields over 90% of estimates within ±3 brpm versus leading methods. The AMTC-derived RR constrains a fast iterative filtering (FIF) decomposition of the best subcarrier (chosen by respiratory spectral energy). This setup enables accurate breathing waveform reconstruction with a 178% higher mean absolute correlation to the chest-belt ground truth than previous CSI-based approaches. Evaluation was limited to a single curated 20-subject overnight polysomnography dataset, leaving slow breathing and home environments untested. Moreover, conjugate multiplication, AMTC, and FIF increase the computational cost, hindering deployment on low-power hardware.

Ge and Ho [37] used commodity Wi-Fi (Huawei WS7002 router) and Raspberry Pi 4B receivers running Nexmon CSI firmware at 5.24 GHz and 80 MHz bandwidth. The CSI from 234 usable subcarriers was separated into amplitude and phase. Each underwent envelope-based preprocessing (amplitude envelope or RMS envelope for phase), which preserves respiratory periodicity. For each subcarrier, a BNR was computed from the FFT within the 10–37 brpm range. Subcarriers with a BNR > 0.7 × max-BNR were retained, and their autocorrelation functions were weighted by BNR and then combined. The RR was estimated from the lag of the first autocorrelation peak. The ground truth came from an iPhone accelerometer strapped to the abdomen, while breathing was paced by a metronome at 15, 20, and 25 brpm, yielding 18 thirty-second files (≈630 k samples). The method achieved a MAE of 0.21 brpm (amplitude) and 0.30 brpm (phase), corresponding to 98.9% and 98.5% accuracy, which is about 6% better than Savitzky–Golay-filtered baselines. However, limitations include single-subject, fixed-rate, and short-duration testing, as well as a narrow respiratory range.

### 2.4. Availability of Datasets for CSI-Based Vital Sign Estimation

CSI-based real world datasets are rapidly emerging for human detection and activity recognition [38,39]. Publicly available datasets that can be used for the training and validation of vital-sign estimation algorithms from CSI data are scarce. A complete dataset should comprise CSI data recorded using a device compliant with modern IEEE 802.11 communication standards. The data should be accompanied by reference vital-sign estimates, which are recorded using established medical equipment, to allow for accurate comparisons between the RR estimates and clinical reference values. The recording time should be sufficiently long and comprise multiple subjects with a wide range of RR values.

The dataset used by *BreathTrack* [29] contains eight subjects only. The reference vital signs have low variability. All reference RR values varied between 12–20 breaths per minute (brpm). These were calculated by asking the subjects to count their own breaths or by following a metronome frequency. Neither of these methods were validated, as the subjects were likely to incur counting errors or follow the breathing metronome inaccurately.

The vital sign monitoring system proposed by Liu [31] was evaluated using a respiratory dataset from only six subjects, with limited variability in the RR (all reference values ranged between 12 and 18 brpm), and metronome-guided breathing was used as the ground truth, which is imprecise. These limitations undermine the authors’ claim, due to a reported MAE of less than 0.2 brpm, of achieving high accuracy. Furthermore, the wireless access point had to be placed within 2 m of the receiver, restricting the system’s practical applicability.

The dataset used to validate the ResBeat system [40] used a belt sensor attached to the subjects to compute the reference values. The belt sensor output, which measures the expansion of the chest, was processed using the same algorithms that process the CSI data to generate RR estimates. By doing so, similar estimation errors will be present in both the reference RR values and the RR estimates from the CSI data. This will likely lead to an increase in the system inaccuracy when validating against other established medical devices.

The eHealth CSI study [39] recently built a large public Wi-Fi CSI dataset to enable contactless vital-sign and activity monitoring. Wi-Fi CSI was collected in a 3 × 4 m room from 118 participants (88 male, 30 female; ages 18–64), each performing 17 activities (e.g., sitting, standing, lying, and walking) for 60 s per position. During each session, participants wore a Samsung Galaxy Watch 4, which logged the heart rate (HR) and RR to provide reference data, while CSI packets were captured via a Raspberry Pi 4B and Wi-Fi router (5 GHz, 80 MHz bandwidth). Empty-room CSI was also recorded to characterise the baseline channel. The dataset includes CSI signals, smartwatch HR/RR values, and participant phenotype data (age, gender, and BMI) in order to support the research on non-invasive HR/RR estimation, human presence, and activity detection from Wi-Fi signals. Key limitations include reliance on smartwatch measurements instead of medical-grade devices as the ground truth and the absence of a strict paced-breathing protocol (the participants only followed brief natural or alternate breathing instructions).

Table 1 presents a structured comparison of *VitalCSI* against representative Wi-Fi CSI-based respiratory monitoring systems, highlighting the differences in sensing principles, hardware complexity, monitoring range, accuracy, and deployment constraints.

## 3. Methods

### 3.1. Wi-Fi Packet Interception System

We developed *VitalCSI*, a data collection system, to facilitate the recording of CSI datagrams from Wi-Fi data packets. Our system runs on a Raspberry Pi 4B single board micro-computer with 4 GB RAM, a Broadcom BCM2711 CPU with an ARM A72 core, and a bcm43455c0 Broadcom single-antenna Wi-Fi chipset. It expands on the open-source project Nexmon CSI [41]. We modified the firmware of the Wi-Fi chipset to enable continuous CSI capture. This places the wireless network interface card (NIC) in monitor mode, whereby it can intercept data packets in the wireless local area network (WLAN). Our software extracts and records the CSI data from the intercepted data packets.

**Figure 1:**
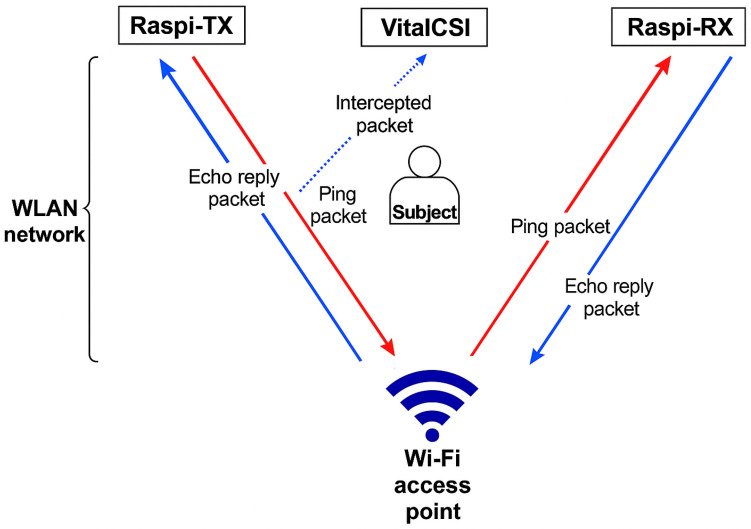
Overview of the proposed CSI system. Ping packets were sent from the Raspi-TX to the Raspi-RX computer via the Wi-Fi access point (red arrows). Echo reply packets were sent by Raspi-RX (blue arrows). The *VitalCSI* computer intercepted these data packets and extracted the CSI data.

We used two additional computers to create traffic on the wireless network, and both computers had the same specifications as *VitalCSI*. Raspi-TX generated traffic by executing the standard Linux ping command to Raspi-RX at a rate of 50 packets per second. The Raspi-RX computer answered the ping requests with echo reply packets (red and blue arrows in Figure 1).

The subject was placed between the Wi-Fi access point and the *VitalCSI* computer. We used a standard ASUS-AC86U (Asus, Taiwan) to provide a Wi-Fi link for the data collection system. This device is an off-the-shelf, commercially available wireless access point. It is compatible with the high-speed IEEE 802.11ac standard. The ASUS-AC86U uses four antennas to transmit Wi-Fi data. We configured the Wi-Fi access point with an 80 MHz bandwidth, providing 256 subcarriers. The transmission channel was set to 36, which corresponds to a central frequency of 5180 MHz. No specialised software was installed on the Wi-Fi access point; as such, it retained its original function as a router, providing WLAN access to multiple devices.

Our setup requires only one computer, *VitalCSI*, for CSI packet collection. The Raspi-TX and Raspi-RX units simply generated consistent network traffic and can be replaced by ordinary Internet-connected computers.

### 3.2. Experimental Setup

#### 3.2.1. Study Design

**Figure 2:**
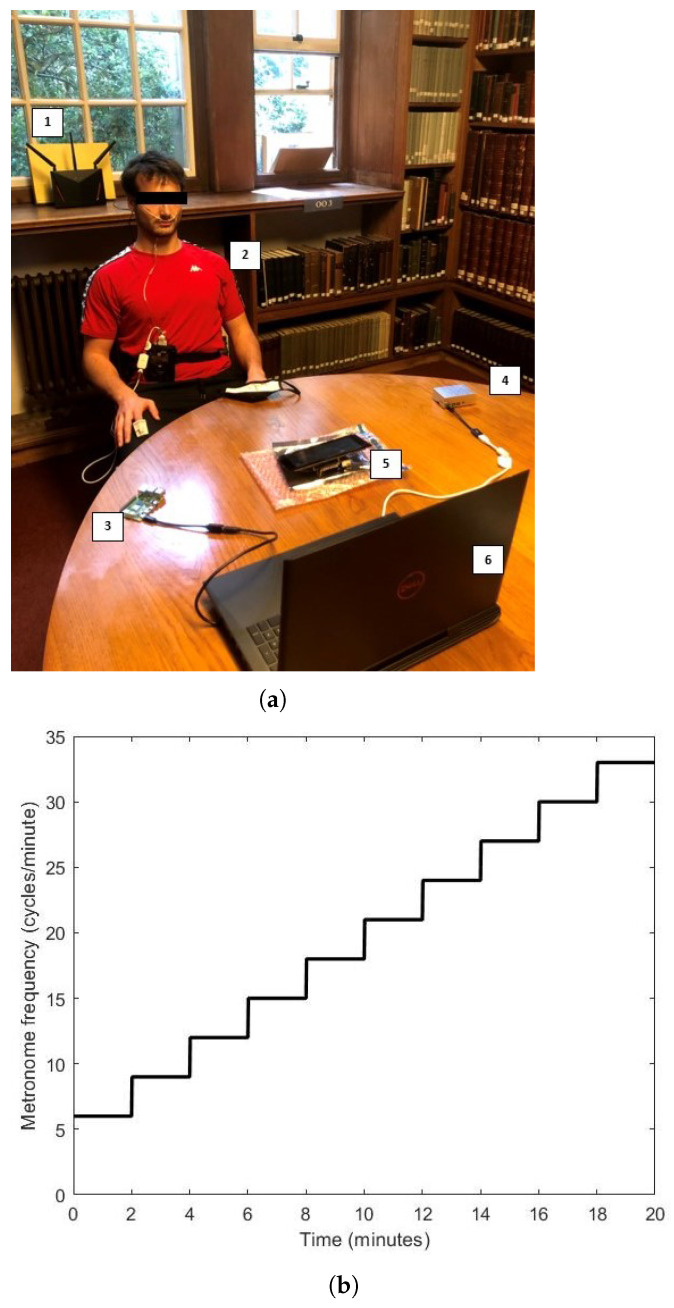
(**a**) A subject being recorded during the experimental study. 1: ASUS-AC86U router; 2: subject with the pulse oximeter and nasal pressure cannula attached; 3: Raspi-TX transmitter; 4: Raspi-RX receiver; 5: *VitalCSI* recorder; and 6: Controller PC. (**b**) Recording protocol guided by a metronome. A step increase of 3 brpm occurred every 2 min. The metronome frequency started at 6 brpm and went up to an upper limit of 33 brpm.

We used *VitalCSI* to record the data from 15 university level athletes. Data was recorded over a two-week period in an isolated room. The subjects were sat on a chair between *VitalCSI* and the Wi-Fi access point, at a distance of approximately 1 m from both devices. The Raspi-TX and Raspi-RX computers were placed 0.5 m on either side of *VitalCSI*. All devices were placed on a table at approximately similar height levels to the ground. Figure 2a shows a subject at the start of the recording session.

We developed a metronome software in MATLAB R2021b (MathWorks, Natick, MA, USA) to help guide the subject’s breathing. We installed the software on a laptop and placed it approximately 1.2 m in front of the subject. A controller PC was used to initiate the CSI recording and display a metronome to help guide the subject’s breathing. Each subject was recorded for 20 min. During this time, *VitalCSI*, a pulse oximeter, and an airflow sensor recorded physiological vital signs. Since these data recording methods are susceptible to movement artefacts, the subjects were instructed to remain seated and stationary at all times. They were also asked to breathe through their nostrils to allow the airflow sensor to record respiratory signals. Only the study participant was present in the room while recording the study data. The metronome guiding the subject respiratory rate began at 6 cycles per minute and increased by 3 via a step function every 120 s, until the meter reached 33 cycles per minute (see Figure 2b).

We used the BlackShadow multi-signal recording module (Stowood Scientific Instruments Ltd., Oxfordshire, UK), strapped around the waist of the subject (see Figure 2a), to record the reference physiological data. Subjects were asked to breathe through their nostrils. A plastic nasal flow pressure cannula (Stowood Scientific Instruments Ltd.) was used to record a respiratory signal from which the reference respiratory rates could be computed. The nasal flow sensor was recorded at a rate of 32 Hz. A pulse oximeter sensor (Masimo Corp, Irvine, CA, USA) was attached to the index finger of the participant’s right hand. The manufacturer provided estimates of HR and blood oxygen saturation (SpO_2_) at a rate of 1 Hz.

#### 3.2.2. Computing the Reference RR

The Blackshadow device used in this study records the respiratory waveform from the airflow sensor attached to the subject’s nostrils. However, it does not compute the RR estimates. When the subject inhaled and exhaled, the local pressure in the nasal flow pressure cannula in the nostrils decreased and increased, respectively. Pressure changes in the nostrils resulted in a waveform correlating to the subject’s breathing, from which the RR could then be computed. We computed the reference RR from the airflow data at a frequency of 1 Hz. Following the work of Chaiculee [42], we applied an 8th-order IIR bandpass filter to remove the frequency components outside of the physiological range of breathing (taken to be between 5–35 brpm in our experimental setup). We then applied a 30 s sliding window with a 1 s step. Finally, we used a peak detection algorithm to estimate the RR for each window.

We followed the peak detection technique proposed by Bettermann et al. [43] and validated by Schafer et al. [44]. The algorithm begins by finding the maxima and minima points of the filtered airflow data. The absolute vertical differences between the adjacent maxima and minima are then determined and the third quartile ${\hat{Q}}_{3}$ of these values is calculated. We used a recursive algorithm to discard all of the peaks that had a vertical separation between them and the nearest trough that was less than 0.3 ${\hat{Q}}_{3}$. The resultant peaks and troughs were associated with the underlying respiratory process. The RR was computed for each 30 s time window by counting the number of peaks and dividing by the time between the first and last peak.

Since the subjects were unlikely to have followed the metronome at all times during the 20 min sessions, the metronome could only be used as an approximate indicator of the underlying RR. To avoid periods where the RR reference estimates did not represent the RR of the subject, all of the periods of recording, where the difference between the airflow estimates and metronome was greater than 4 brpm, were removed from the analysis of the results. This accounted for only 7.4% of the dataset.

### 3.3. Wi-Fi Channel State Information

To estimate the wireless channel, Wi-Fi systems transmit sounding frames containing predefined training symbols. As these frames propagate through the environment, they experience changes in phase and amplitude due to reflection, scattering, and absorption. The receiver compares the incoming signal with its known reference to estimate the channel propagation matrix, which characterises the transformation of the transmitted signal by the wireless medium [45]. This matrix is then used to optimise MIMO transmission strategies, such as beamforming.

The channel propagation matrix, H, describes how the environment alters the transmitted signal, x, to produce the received signal, Y, via the following equation:

(1) Y=Hx+n,

where n represents additive Gaussian noise. In a MIMO-OFDM system with *M* transmit antennas, *N* receive antennas, and *K* subcarriers, the channel matrix is a three-dimensional complex tensor: $H\in C^{M\times N\times K}$. Each entry $h_{i,j,k}$, corresponding to the ith transmit antenna, jth receive antenna, and kth subcarrier, is computed as follows:

(2) $$h_{i,j,k}=\sum_{n=1}^{N}a_{n}\left(t\right)e^{-j2\pi f\tau_{n}\left(t\right)},$$

where $a_{n}\left(t\right)$ is the amplitude attenuation coefficient, *f* is the transmission frequency, and $\tau_{n}\left(t\right)$ is the propagation delay at time *t*. The resulting channel state matrix H, known as CSI, encodes the amplitude and phase response of the environment with high precision.

### 3.4. Respiratory Rate Estimation from Wi-Fi Signals

Respiratory effort causes displacement of the chest and abdominal wall, which changes the distance between the chest of the subject and a wireless receiver (such as a Wi-Fi router). This results in changes to the amplitude and phase of the wireless signals reflected off the subject’s body.

**Figure 3:**
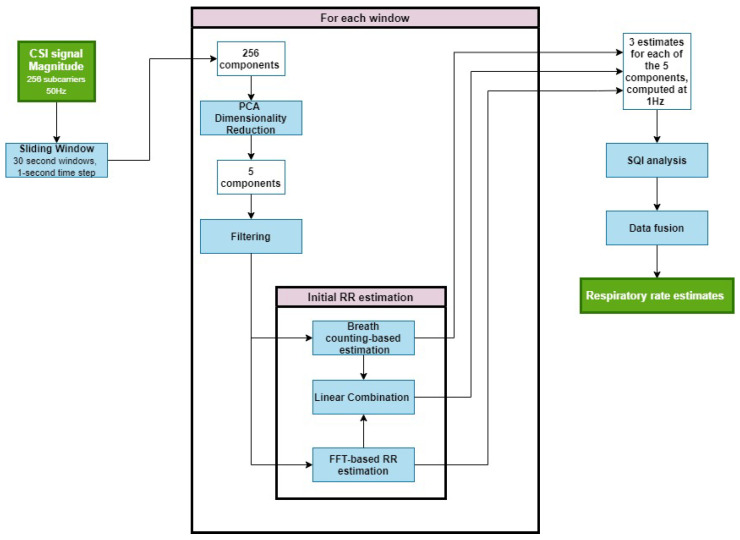
Pipeline for respiratory rate (RR) estimation from the Wi-Fi CSI data. CSI signal magnitudes (256 subcarriers at 50 Hz) were segmented into 30 s sliding windows with a 1 s step to allow RR estimation at 1 Hz. Principal component analysis (PCA) reduces each window to five components, which are bandpass filtered to retain the 6–33 brpm frequency range. For each component, the RR is estimated using two complementary methods—spectral peak detection in the frequency domain and breath counting in the time domain—and these are combined via a multidimensional Kalman filter. Signal-quality (SQI) analysis and data fusion then yield a single robust RR estimate per window.

The RR estimation process is shown in Figure 3. First, we extracted the magnitude of the CSI data, yielding a 256-dimensional time series. A 30 s sliding window with a 1 s step was applied, allowing RR estimates to be calculated at 1 Hz. We then performed PCA to reduce the dimensionality, extracting five components per window and filtering each to retain the frequency content within the experimental range (6–33 brpm). Spectral peak estimation in the frequency domain and breath counting in the time domain were both applied to each component, producing two initial RR estimates per component. Finally, a multidimensional Kalman filter was used to merge these into a single estimate per window. The resultant RR estimates were output at 1 Hz.

#### 3.4.1. Dimensionality Reduction

The majority of previous methods perform RR estimation directly from a subset of all subcarriers. This is often ineffective as CSI signals are frequently affected by motion artefacts and other sources of noise.

**Figure 4:**
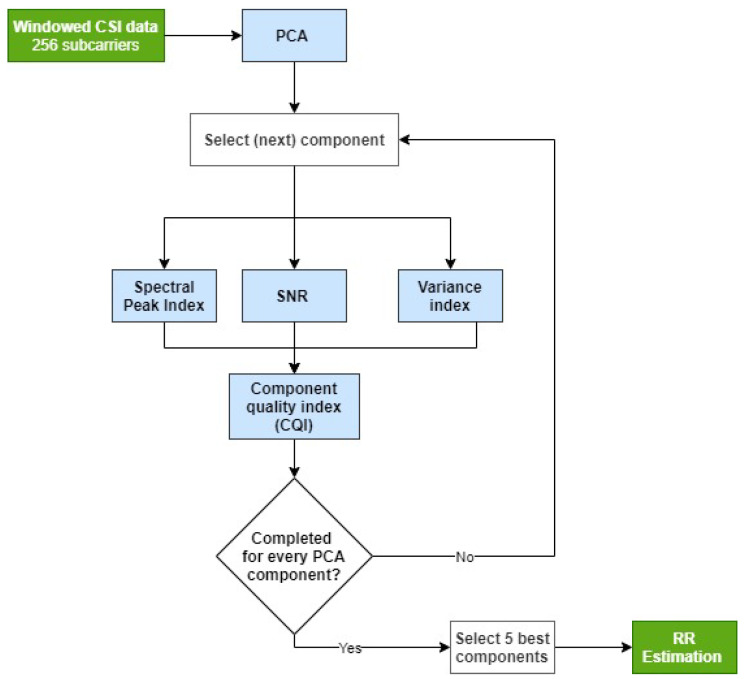
Overview of the process to select the Wi-Fi subcarriers for respiratory rate estimation. The input is a 30 s window of CSI data containing 256 subcarriers. Five PCA components are selected for respiratory rate estimation.

Figure 4 illustrates our proposed method for selecting subcarriers that contain the respiratory signal. The algorithm reduces the original 256-dimensional CSI data to a lower-dimensional space that retains the respiratory signal while minimising noise and motion artefacts. This reduction also decreases the computational cost of subsequent estimations compared to using all 256 original subcarriers.

**Figure 5:**
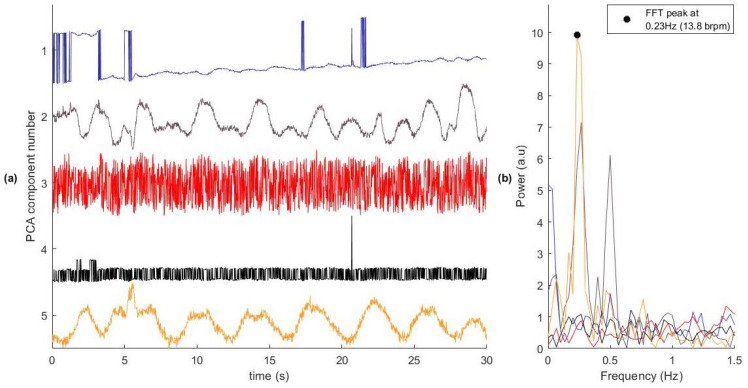
The first five PCA components of a CSI data window. (**a**) The time series of each component. (**b**) The spectral properties of each component. Component Number 2 and 5 contain a clear respiratory signal. Components 1, 3, and 4 contain little useful information for the analysis of the RR and should be discarded. The reference respiratory rate for this window was 0.29 Hz (17.1 brpm). In practice, all 256 PCA components were assessed.

We applied PCA to transform the original 256-dimensional CSI time series into 256 orthogonal principal components (PCs). Some of these uncorrelated signals contained RR information suitable for RR estimation. In a typical window, only a few components carried meaningful information, as shown in Figure 5, where only two of the five components displayed respiratory signals. To address this, we used three criteria to identify a subset of PCs with the strongest respiratory content: variance-based selection, SNR, and spectral peak location. We then combined the three indices to compute the component quality index (CQI), retaining the components with the highest CQIs for RR estimation.

*Variance-based selection*—RR signals typically account for a significant proportion of the signal variance and are often concentrated in a subset of PCs. To identify the informative components, a variance index $\Gamma_{\sigma_{i}}\left(w\right)$ was computed for each component *i* in a data window *w* as follows:

(3) $$\Gamma_{\sigma_{i}}\left(w\right)=\left\{\begin{array}{cc} 1 & \mathrm{if}\;\frac{\sigma_{i}}{\sigma_{\mathrm{total}}}\geq \sigma_{\mathrm{threshold}}\\ 0 & \mathrm{otherwise} \end{array}\right.,$$

where $\sigma_{i}$ is the variance of the $i^{\mathrm{th}}$ subcarrier, and $\sigma_{\mathrm{total}}$ is the total variance across all components.

**Figure 6:**
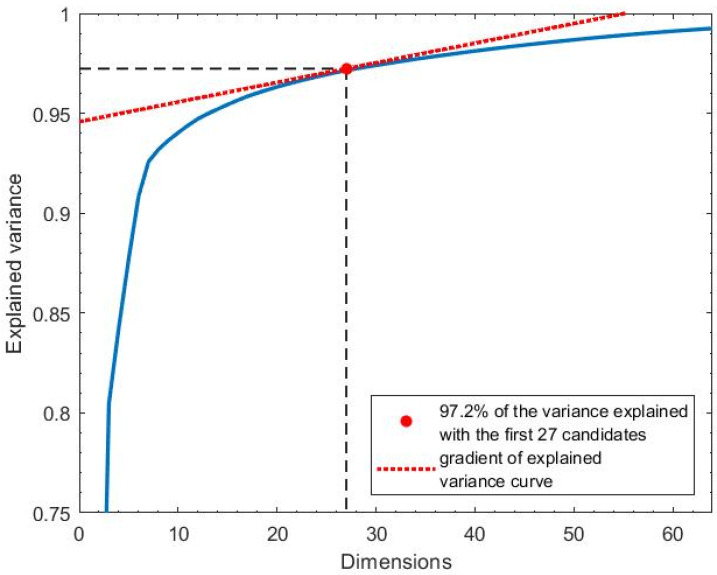
The explained variance was the total variance of the original signal that remained after retaining the first *m* components. It was plotted as a function of the number of PCA components retained. In this illustrative example, 27 principal components accounted for 97.2% of the total signal variance.

The variance threshold $\sigma_{\mathrm{threshold}}$ was selected so as to retain the sufficient signal variance while discarding components that are unlikely to contain clean respiratory information. A common method for variance-based PCA selection is to use the cumulative variance plot, identifying an “elbow point” beyond which additional components contribute little to the overall variance. We selected $\sigma_{\mathrm{threshold}}$ adaptively by identifying the smallest number of PCs at which the gradient of the cumulative variance curve fell below 1% additional variance per component. This approach allows for dynamic adjustment based on the distribution of variance in each data window. This process is illustrated in Figure 6 for a sample window, where $\sigma_{\mathrm{threshold}}=0.972$. Here, 27 components are assigned a $\Gamma_{\sigma_{i}}$ value of 1. On average, 29 components per window were selected ($\Gamma_{\sigma_{i}}=1$), with a standard deviation of 3.2.

*Signal-to-noise ratio (SNR)*—An optimal PCA component contains periodic respiratory signal and no additional motion artefacts or high frequency noise. The frequency domain of this optimal component has the majority of its power in the physiological RR range, which is taken to be between 5–35 brpm (0.083–0.583 Hz) [28]. To select components that are similar to the optimal component, the SNR index, $\Gamma_{SNR_{i}}\left(w\right)$, is calculated for each component, *i*, from the windowed CSI data using the following equation:

(4) $$\Gamma_{SNR_{i}}\left(w\right)=\frac{\sum_{f=f_{1}}^{f_{2}}FFT\left(f_{i}\right)}{\sum_{f=1}^{N}FFT\left(f_{i}\right)},$$

where $f_{1}$ and $f_{2}$ represent the FFT bins corresponding to the valid frequency range for RR of 5–35 brpm (0.083–0.583 Hz), respectively, and $\Gamma_{SNR_{i}}\left(w\right)$ ranges from 0 to 1. Higher values indicate that most of the component’s frequency content lay within the valid RR range.

*Spectral peak selection*—Some principal components capture low-frequency trends in the CSI signal, such as the 1/f noise, which may be mistaken for valid respiratory signals. These components often carry high variance and exhibit elevated SNR, leading to high values for both $\Gamma_{\sigma_{i}}$ and $\Gamma_{SNR_{i}}$, despite lacking physiological relevance. To address this, a spectral peak index $\Gamma_{{\mathrm{peak}}_{i}}$ was introduced:

(5) $$\Gamma_{{\mathrm{peak}}_{i}}=\left\{\begin{array}{cc} 1 & \mathrm{if}\;f_{{\mathrm{peak}}_{i}}\geq f_{\min}\\ 0 & \mathrm{otherwise} \end{array}\right.,$$

where $f_{{\mathrm{peak}}_{i}}$ is the frequency of the spectral peak, and $f_{\min}$ is the minimum physiological RR, which was set to 5 brpm.

The final component quality index (CQI) was computed by combining the three binary indices detailed above:

(6) $$\mathrm{CQI}=\Gamma_{\sigma_{i}}\cdot \Gamma_{SNR_{i}}\cdot \Gamma_{{\mathrm{peak}}_{i}}.$$

We computed the CQI for each component to assess the strength and consistency of the respiratory signal content. On average, components ranked lower than the 5th showed CQI values below 0.05, indicating minimal respiratory contribution. Therefore, only the five components with the highest CQIs were selected for downstream RR estimation.

#### 3.4.2. Filtering

**Figure 7:**
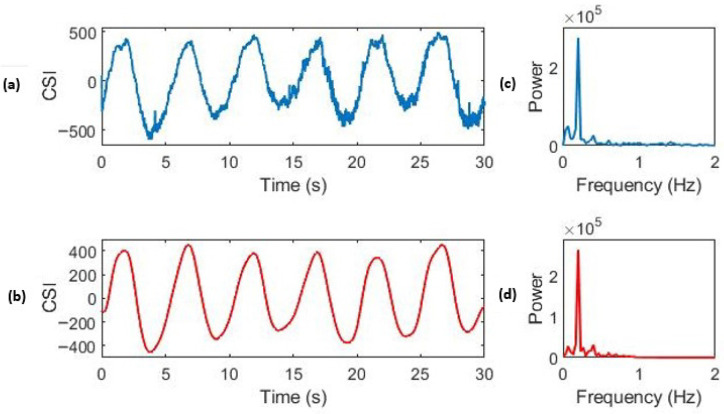
Effect of IIR filtering on high-quality CSI respiratory data. (**a**) The time domain of the unfiltered CSI data. (**c**) The frequency domain of the unfiltered data. (**b**) The time domain of the CSI data after filtering, showing a clear respiratory waveform. (**d**) The frequency domain of the CSI data after filtering. The reference respiratory rate for this window was 12.0 brpm (0.20 Hz).

The selected PCs contain both low- and high-frequency noise from sources such as subject movement and sensor artefacts. To remove frequency components outside the physiological breathing range (5–35 brpm), a digital high-pass and low-pass filter were applied sequentially to the time series, as described in Section 3.2.2. The effect of this filtering is shown in Figure 7, which displays a window of the original PCA component.

#### 3.4.3. Respiratory Rate Estimation

We used two different methods to estimate the RR from the filtered PCA components (FPCs)—breath counting in the time domain and FFT in the frequency domain. We then used the average of the estimates from these two methods.

*Breath counting*—Breath counting is performed by implementing a box slope sum function (BSSF) algorithm, which was first developed by Zong et al. [46] and then refined by Villarroel [47]. This algorithm determines the onset of peaks within a time series before implementing an adaptive filtering method to remove spurious peaks. Breath counting is the simple process of using these breath onsets to detect the RR of a filtered PCA component, $RR_{\mathrm{breath}(FPC)}$, in breaths per minute (brpm) for a given window. It is calculated as follows:

(7) $$RR_{\mathrm{breath}(FPC)}=\frac{N_{\mathrm{onsets}}-1}{t_{{\mathrm{onset}}_{\mathrm{final}}}-t_{{\mathrm{onset}}_{\mathrm{start}}}}\cdot 60,$$

where $N_{\mathrm{onsets}}$ is the number of onsets in the window, $t_{{\mathrm{onset}}_{\mathrm{final}}}$ is the timestamp of the final onset, and $t_{{\mathrm{onset}}_{\mathrm{start}}}$ is the timestamp of the first onset.

*Respiratory frequency*—A Hann window is applied to the signal before the FFT is computed, and the frequency of the largest value is taken as the breathing rate estimate. The FFT has a frequency resolution given by 1/T, where *T* is the length of the window in seconds. Since T=30s, the frequency resolution of the estimates, $RR_{FFT}$, is 0.033 Hz (2 brpm). This has implications in the accuracy of the predictions from this method.

An initial combined estimate ($RR_{\mathrm{combined}}$) was computed for each projection as the mean of the above two methods:

(8) $$RR_{\mathrm{combined}}=\frac{RR_{\mathrm{peak}}+RR_{FFT}}{2},$$

where $RR_{FFT}$ and $RR_{\mathrm{peak}}$ are the estimates from the FFT and breath counting methods, respectively.

#### 3.4.4. Signal Quality Index (SQI)

The SQI aims to encapsulate information about the reliability of estimations by considering the properties of the time series that generated the result. A signal that greatly resembles a respiratory waveform is likely to generate accurate results, and so its SQI should be large. The SQI for an FPC, *i*, is bounded between 0 and 1 and computed using the following equation:

(9) $$SQI_{i}=\Gamma_{SNR_{i}}\cdot \Gamma_{Agree_{i}}\cdot \Gamma_{SPI_{i}},$$

where $\Gamma_{SNR_{i}}$ is the SNR of the component calculated before the filtering step using the method outlined in Section 3.4.1, $\Gamma_{Agree_{i}}$, which is the estimation agreement factor (EAF), and $\Gamma_{SPI_{i}}$ is the spectral purity index (SPI). These are described below.

*Estimation agreement factor*—The estimation agreement factor, $\Gamma_{Agree_{i}}$, uses the absolute agreement between the estimates obtained from the FFT method, $RR_{FFT_{i}}$, and the breath counting method, $RR_{Peak_{i}}$, which is outlined in Section 3.4.3. It is calculated for the $i^{\mathrm{th}}$ PCA component as follows:

(10) $$\Gamma_{Agree_{i}}=\left\{\begin{array}{cc} 1, & \mathrm{if}\;|RR_{FFT_{i}}-RR_{breath_{i}}|\leq 4\;\mathrm{brpm}\\ 0, & \mathrm{otherwise} \end{array}\right..$$

During a period of high-quality signals, it is expected that both methods yield similar values. Therefore, the estimated SQI is set to 0 if the estimates differ by more than 4 brpm. This threshold is chosen since we expect regular steps of 3 brpm in the signal due to the discrete changes in the metronome frequency that guided the subject’s breathing rate.

*Spectral Purity Index (SPI)*—High-quality respiratory signals typically exhibit a sinusoidal waveform, with RR as the fundamental frequency. To identify components with this characteristic, we used the SPI, which is based on the method proposed by Nemati et al. [48]. This is calculated using the nth order spectral moment $\bar{\omega_{n}}$ of the spectral power $S(e^{j\omega })$, which is computed as follows:

(11) $$\bar{\omega_{n}}=\int_{-\pi }^{\pi }\omega^{n}S_{x}\left(e^{j\omega }\right)d\omega .$$

The spectral purity of a PCA component *i*, $\Gamma_{SPI}\left(i\right)$, is computed as follows:

(12) $$\Gamma_{SPI}\left(i\right)=\frac{{\bar{\omega_{2}}}^{2}\left(i\right)}{\bar{\omega_{0}}\left(i\right)\cdot \bar{\omega_{4}}\left(i\right)}.$$

The SPI takes a value of 1 for signals with a single dominant frequency component, and it approaches 0 for signals resembling Gaussian white noise.

#### 3.4.5. Data Fusion

The RR estimation process yields five RR estimates, one for each of the five PCA components extracted from Wi-Fi CSI data. We used a multidimensional Kalman filter (Kalman ND) to fuse the estimates and obtain the final RR estimate per time window. This resulted in a complete RR time series for each recording session. Kalman ND is well suited to this setting because it explicitly accounts for the differing reliability of each PCA-derived RR estimate, where multiple noisy sources are dynamically weighted and fused to produce a more stable and accurate RR estimate than any single component alone. This approach is motivated by the work of Li [49] and Tarassenko et al. [50], who demonstrated that Kalman ND is effective for estimating physiological vital signs from multiple sources.

The technique first applies one-dimensional Kalman filtering to each PCA component *i*, and it then computes the ith RR estimate, $RR_{i}$, as a weighted average of the RR estimates from each available source:

(13) $$RR_{i}=\sum_{k=1}^{n}\left(\frac{\left(\prod_{\begin{array}{c} i=1\\ i\neq k \end{array}}^{n}\sigma_{i}^{2}\right)}{\sum_{i=1}^{n}\left(\prod_{\begin{array}{c} j=1\\ j\neq i \end{array}}^{n}\sigma_{j}^{2}\right)}\cdot RR_{k}\right),\;k=1,2,\ldots ,n,$$

where *k* is the number of prediction sources being fused, and $RR_{k}$ is the RR estimate for the corresponding source. The residual value σ is calculated using the following:

(14) $$\sigma_{i}^{2}={\left(\frac{r_{i}}{SQI_{i}}\right)}^{2},\;k=1,2,\ldots ,n,$$

where $SQI_{i}$ is the signal quality index of the estimation source, $r_{i}$ is the residual error of the Kalman filter, and *k* is the measurement number (up to 5 for each of the input RR estimates).

### 3.5. Evaluation Metrics

To validate the CSI-based RR estimates, the reference respiratory rates were computed from the airflow data, as described in Section 3.2.2. The agreement between the CSI-derived and reference RR values was quantified using multiple complementary metrics.

*Mean Absolute Error (MAE):*

$$\mathrm{MAE}=\frac{1}{N}\sum_{i=1}^{N}\left|{\hat{R}}_{i}-R_{i}\right|,$$

where ${\hat{R}}_{i}$ and $R_{i}$ are the estimated and reference respiratory rates for window *i*, and *N* is the total number of windows.

*Root Mean Squared Error (RMSE):*

$$\mathrm{RMSE}=\sqrt{\frac{1}{N}\sum_{i=1}^{N}{\left({\hat{R}}_{i}-R_{i}\right)}^{2}}.$$

The RMSE highlights larger deviations more strongly than MAE.

*Coefficient of determination ($r^{2}$):* this metric quantifies the strength of the linear relationship between the estimated and reference RR values.

*Percentage within 2 brpm ($RR_{2\;\mathrm{brpm}}$):* this is the proportion of estimates that fall within ±2 brpm of the reference values.

*Bland–Altman analysis:* this is used to assess the systematic bias and limits of agreement by plotting the difference between the estimated and reference RR against their mean.

## 4. Results

### 4.1. Cohort Description

**Figure 8:**
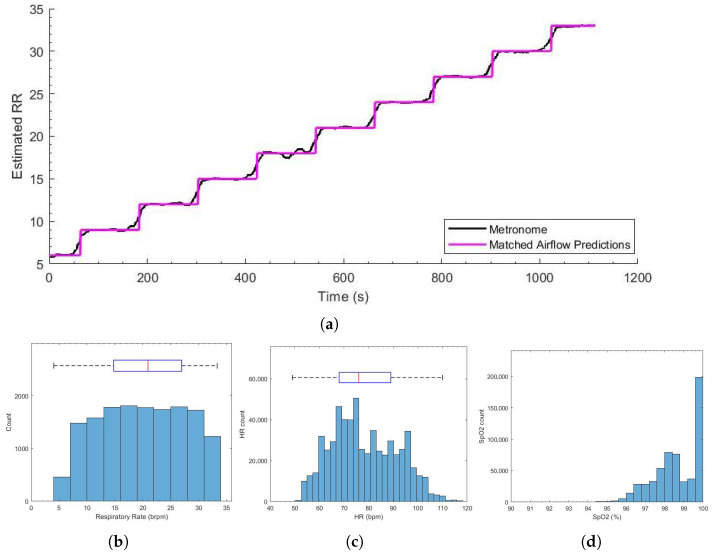
(**a**) The metronome frequency (black) and the reference respiratory rate computed from the airflow data (purple) for the entire recording session. (**b**) The respiratory rate estimates from the airflow data. (**c**) The heart rate distribution. (**d**) SpO_2_ estimates from the Masimo pulse oximeter.

We recruited 15 healthy subjects between 18 and 60 years in age. Table 2 shows the demographics of the recorded population. Notably, 73.33% of participants had experience in competitive sports (40% rugby or 6/15 participants, 20% football or 3/15 participants, and 13.33% lacrosse or 2/15 participants), providing a cohort representative of physically active individuals while maintaining the controlled setting required for this proof-of-concept study. The total recording time from all sessions was 300 min. As can be seen in Figure 8a, the reference RR computed from the airflow data followed the metronome frequency closely. The MAE between the two estimates was 0.8 brpm, and 92.6% of estimations were within 4 brpm of each other (with an RMSE of 1.80 brpm).

Figure 8b–d shows the histograms of the vital sign distributions. The large RR standard deviation was a result of the respiratory metronome used to create variation in the subject vital signs. The SpO_2_ and HR distributions were within the expected range for healthy volunteers.

### 4.2. Respiratory Rate Estimation

**Figure 9:**
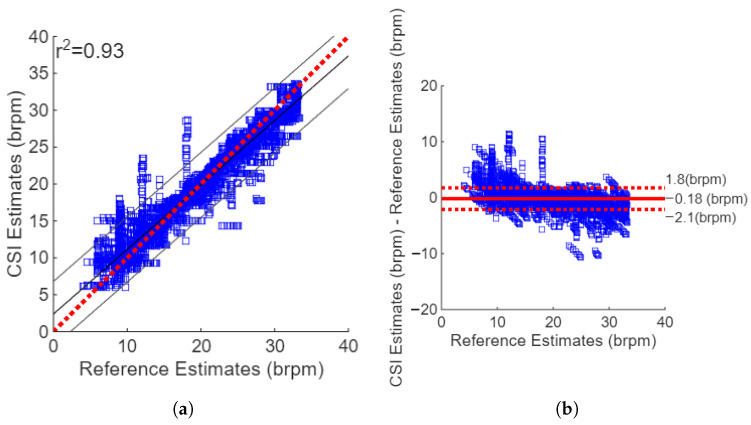
The agreement between the Wi-Fi respiratory rate estimates and the reference values. (**a**) Correlation plot showing low sensor bias; $r^{2}=0.89$. (**b**) Bland–Altman plot showing the CSI-reference differences versus their mean. Sensor bias is low (−0.18 brpm).

The RR estimates from the Wi-Fi data yielded an MAE of 1.23 brpm and an RMSE of 2.01. The correlation plot shown in Figure 9a shows strong positive agreement between the estimates and reference values, with a squared Pearson’s correlation coefficient $r^{2}$ of 0.93. The Bland–Altman plot for Kalman ND filtering and the combination of the FFT and breath count method, as shown in Figure 9b, revealed a small sensor bias, with a mean difference between the reference values and CSI estimates of −0.18 brpm. The difference between the estimates and reference values were well bounded with 95% confidence intervals of −2.1 and 1.8 brpm. There were vertical lines in the Bland–Altman plot that were found at an RR of 9, 12, and 18 brpm. These are caused by the step changes in the metronome frequency used to guide the subjects’ breathing, and they suggest that the subjects followed the metronome.

**Figure 10:**
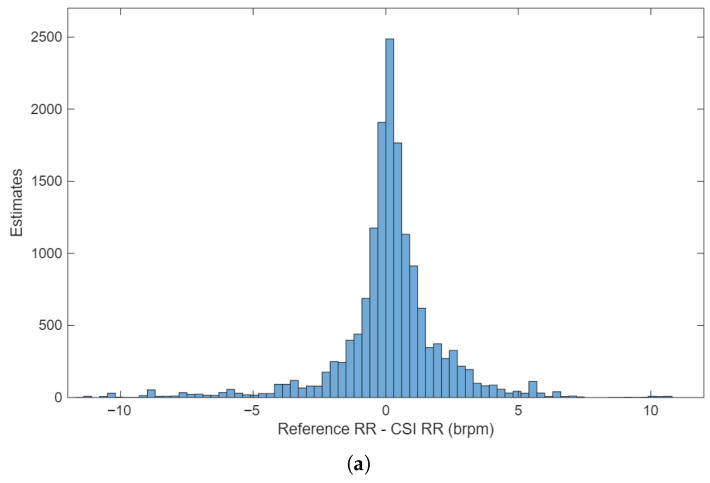

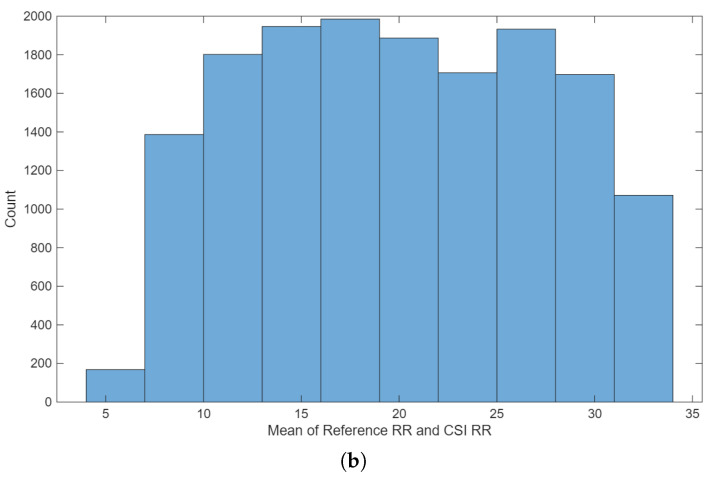
(**a**) The distribution of the RR values computed as the mean of the Wi-Fi and reference estimates. (**b**) The distribution of the differences between the Wi-Fi and reference RR values.

The histogram of errors, as shown in Figure 10a, reveals that the errors were normally distributed. The mean of the reference and Wi-Fi-based estimates for the corresponding windows revealed that almost all the RR values were found in the range of 6–33 brpm, as shown in Figure 10b. This was expected since this was the range of the metronome used to guide the subject RR.

**Figure 11:**
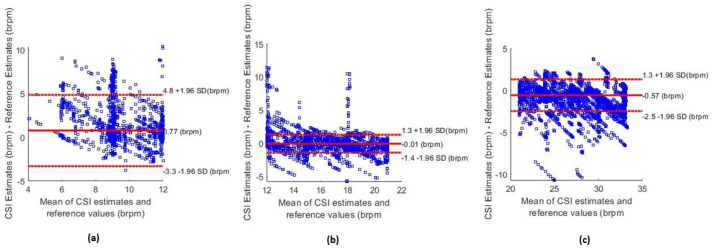
Bland–Altman plots for different respiratory rate values. (**a**) Estimates below 12 brpm. (**b**) Estimates in the normal respiratory rate range between 12–21 brpm. (**c**) Estimates above 21 brpm.

Figure 11 shows the Bland–Altman plot for the Kalman ND filtering and the combination of the FFT and breath count method, which was segmented based on the value of the estimated RR. Figure 11a shows the estimates that were obtained when the mean of the reference RR values and estimates was less than 12 brpm. Figure 11b,c show the segmented Bland–Altman plots for 12–21 brpm and over 21 brpm, respectively. These plots revealed that the accuracy of estimates was lower for periods of breathing under 12 brpm than for a higher RR.

## 5. Discussion

The high level of agreement between the airflow estimates and the metronome frequency shows that the proposed algorithm for the reference RR estimation performed reliably, with the metronome serving as a useful sanity check.

The results reveal a high level of agreement between the RR estimates from Wi-Fi and those from established methods in our controlled environment. The final algorithm developed obtained a low MAE of 1.20 brpm and showed strong positive correlation with the airflow-based estimations ($r^{2}=0.93$). This effectively demonstrates that accurate RR estimation can be feasibly performed in a controlled environment. The high accuracy of our system can also be attributed to PCA-based dimensionality reduction and the choice of the Kalman ND filter to enable fusion from multiple principal components.

The accuracy of our system is comparable to established contact-based monitoring systems, such as the Masimo Corp acoustic monitoring sensor, which gained FDA approval with a stated MAE of 1 brpm. It is also similar to contactless RR estimation techniques in clinical settings. Villarroel [47] proposed a camera-based system to measure the patient RR as they underwent haemodialysis treatment, the MAE of which was approximately 1.8 brpm.

The algorithm had a marginally reduced performance during periods of recording when the reference RR was low. The MAE of the estimates when the reference RR was below 12 brpm was 1.97 brpm (compared to 1.20 brpm overall). This is also evident in the Bland–Altman plot in Figure 11a. This degradation is likely due to the reduced number of respiratory cycles per analysis window and the lower-frequency and lower-amplitude signal components at slow breathing rates, which make accurate spectral and temporal estimation more sensitive to noise and windowing effects. A positive sensor bias of +0.77 brpm was observed, and the 95% confidence intervals were wider than the ranges shown in Figure 11b,c. Low breathing rates are of particular importance as they can be used to detect conditions, such as sleep apnoea [5] and postoperative bradypnea [51]. Improving the accuracy of low RR estimates is, therefore, an important future task.

The efficacy of the filtering methods was limited by the wide range of physiological respiratory rates. This is because the filter cutoff frequencies had to be set far apart to ensure that valid respiratory data were not filtered out. As a result, during periods of low RR, the filter was ineffective at removing certain high-frequency components. Consequently, the time-based RR estimation methods must be more robust to false peaks in the data.

The two RR estimation methods proposed (breath counting and spectral peak) were both effective in predicting the RR from high-quality CSI data. Both methods, however, were negatively affected by the effect of a short sliding window of 30 s. This window length restricted the resolution of FFT methods to just 2 brpm. Since the accuracy of the algorithm was around 1.20 brpm, this low resolution provided a minimum boundary to the overall error. As noted previously, during periods of low RR, there may only be as few as two complete breaths during an entire window. The accuracy of the breath counting methods may be compromised as the noise in the signal will have a great effect on the final RR estimations. Simply increasing the length of the window will increase the accuracy of both RR estimation methods. However, this will reduce the estimation time resolution resulting in the algorithms being slow to react to sudden changes in the breathing rate of the subjects.

CSI-based datasets for activity recognition are increasing [38,39], but there is still a notable lack of publicly available datasets designed specifically for vital-sign estimation. For meaningful evaluation, such datasets must include CSI data captured using modern IEEE 802.11 hardware alongside synchronised clinical ground truth measurements (e.g., respiratory belts, spirometry, or ECG) to permit accurate performance benchmarking. They should also span sufficiently long recordings, multiple participants, and a physiologically broad distribution of respiratory rates. The current scarcity of such datasets remains a major barrier to reproducible research and clinical validation.

While this study focused on a controlled, seated breathing setup to isolate the respiratory component of the Wi-Fi channel, the predominantly athletic profile of participants (73% having played competitive sports, including rugby, football, and lacrosse) highlights a natural translational pathway toward sports and performance science. In such contexts, non-intrusive respiratory monitoring could support longitudinal tracking of recovery, exertion load, and cardiorespiratory adaptation without the burden of wearable sensors.

Beyond the core findings presented above, it is important to consider both algorithmic extensions and deployment-related challenges that were not addressed within the scope of the present study. A method that could potentially provide improved dimensionality reduction is periodic component analysis [52]. This technique utilises the constructive properties of a periodic signal to isolate waveforms from high-dimensional data. Jorge et al. [12] demonstrated that, when applied to multi-channel camera data, the method was able to isolate the respiratory signal in a single component. Therefore, it eliminates the need for *a posteriori* criteria to select components containing the respiratory signal.

Wi-Fi vital-sign monitoring in the home environment presents numerous challenges that fall beyond the scope of this project. These include multi-subject RR estimation, where more than one individual is present near the Wi-Fi router. In such environments, there will also be periods of significant subject motion as individuals move around the space. The accuracy of a Wi-Fi-based vital-sign estimation system must, therefore, be evaluated as the distance between the subject and receiver increases, as well as in conditions where walls or other objects obstruct the signal path. The present study used a fixed recording distance of approximately 1 m between the subject and the Wi-Fi devices to ensure signal stability and to isolate the algorithmic performance in a controlled setting. The effects of longer distances, obstructions, and dynamic positioning remain important open questions for real-world deployment and are left to future work.

## 6. Conclusions

This research proposes *VitalCSI*—a Wi-Fi-based vital sign monitoring system using off-the-shelf commercially available equipment. It provides a continuous, non-intrusive, and privacy-preserving technology for monitoring respiratory rate without requiring line of sight. Our experimental study dataset captured a wide range of physiological values, with the subject breathing rate varying between 6–33 brpm. This is a greater range than other works (typically 12–20 brpm). The dataset contains data from 15 subjects, which is significantly larger than the current published studies, typically six subjects on average. The total length of recording time of our dataset is 300 min.

The accurate reference RR values were recorded using equipment validated in the literature. As a result, the dataset created is an optimal tool to develop and validate CSI-based RR estimation algorithms. *VitalCSI* also records CSI from all 256 subcarriers at a rate of 50 Hz, which is a considerably wider dimension and frequency than systems previously used in the literature. It is the first known project to demonstrate RR monitoring with a single-antenna Raspberry Pi computer as the Wi-Fi data packet scanning system. The RR estimates from the Wi-Fi data revealed a high level of agreement with the values from the reference device.

Future work would benefit from further algorithm improvements. The accuracy of the Wi-Fi-based vital sign estimation system must be assessed as the distance from the subject and Wi-Fi receiver increases, as well as when walls and other objects lie between the subject and the receiver. The RR estimation algorithms should be able to take into account multi-person scenarios and large subject movements. An experimental study should be conducted with the aim of developing a Wi-Fi CSI vital sign dataset in the home environment. This would involve a large number of subjects being constantly monitored in their homes with a combination of the proposed *VitalCSI* system and an established reference RR monitoring device. This device should be minimally invasive and, unlike the method used in our experimental study, be able to monitor subject breathing, regardless of whether they are breathing through their mouth or nose.

The proposed RR monitoring system could be harnessed to detect vital signs during sleep. This would allow important health metrics to be determined, such as the circadian rhythm, movement during sleep, or the diagnosis of sleep apnoea. There is also a great clinical need for a non-contact system that can detect falls in real time and notify emergency services without delay.

Wi-Fi-based RR estimation is a capable solution to meet the growing demand for vital sign monitoring, especially in the home environment. Through the creation of a Wi-Fi monitoring system prototype and contributions to an open-source project, including the development of validated RR estimation algorithms, this work paves the way for future research into the applications of CSI for physiological monitoring. It advances research into scalable contactless RR monitoring solutions that could be seamlessly integrated into the domestic environment.

This preliminary study also lays the groundwork for future applications in athlete monitoring. The inclusion of participants with competitive sporting backgrounds highlights the potential of Wi-Fi-based respiratory sensing to evolve toward real-world, sport-specific contexts, where unobtrusive physiological monitoring is increasingly sought.

## Figures and Tables

**Table 1:** Comparison of Wi-Fi CSI respiratory monitoring systems.

System	Principle	Hardware	Monitoring Range	RR Range	Accuracy	N	Deployment
***VitalCSI*** **(this work)**	CSI magnitude; adaptive PCA and Kalman ND fusion	Commodity Wi-Fi AP (2.4/5 GHz) transmitter, Raspberry Pi receiver	1 m	6–33 brpm	MAE = 1.20 brpm, $r^{2}$ = 0.93	15	Single antenna; controlled setting; strong reference alignment
*BreathTrack* [29]	CSI phase; joint AoA–ToF sparse recovery; FFT peak detection	Intel 5300 NICs (Tx/Rx), 5.31 GHz; Rx antenna array with reference RF chain for phase calibration	2 m	12–20 bprm	Median breath-rate accuracy > 99%	8	Custom Wi-Fi NIC; calibrated multi-antenna setup
Wi-Covid [30]	CSI magnitude; PCA; wavelet spectrogram peak detection	Raspberry Pi 4, Nexmon CSI; Commodity Wi-Fi AP	Not reported	12–24 brpm	Not reported	1	PCA component selection not specified
Liu et al. [31]	CSI magnitude; variance-based subcarrier selection; peak detection with variance-weighted fusion	Commodity Wi-Fi AP + single Intel 5300 NIC device (802.11n, 20 MHz)	2–10 m	12–18 brpm	Mean error < 0.4 brpm	6	Single Tx–Rx pair; sleep-specific scenarios; supports two-person in-bed case
Kontou et al. [32]	CSI magnitude; PCA; ANN frequency estimation	Commodity 5 GHz Wi-Fi router (80 MHz) + Raspberry Pi 4 (Nexmon CSI)	0.3–2 m	6–60 brpm (training range)	Mean frequency error = 1.4–12.2%	1 (20)	Single-subject; static scenarios; ANN trained on synthetic data
WiResP [33]	CSI magnitude; auto-correlation; BNR-weighted subcarrier selection; spectrum enhancement	Commodity 802.11ac Wi-Fi (5.8 GHz, 40 MHz); 2 Tx × 1 Rx; 30 Hz CSI	up to 8 m	10–30 brpm	Median error = 0.8–0.9 brpm; false-alarm rate > 5%	1 (35)	Flexible placement; multi-room operation; LOS/NLOS; motion-aware
Burimas et al. [34]	CSI magnitude; multi-stage filtering; local peak counting	ESP32 microcontrollers (Tx/AP + Rx/STA), 2.4 GHz, 22 MHz	1–2 m	Slow (<12 brpm), normal (<12–16 brpm), fast (>16 brpm)	MAD = 2.60 brpm	8	Controlled environment; ≈5 min recordings; sensitive to parameter tuning, motion
Wi-Breath [35]	CSI magnitude + inter-antenna phase difference; SVM-based signal selection; peak-interval RR estimation	Intel 5300 NIC (3 Rx) + Mi Router 3 (2 Tx), 5 GHz	2.5 m	12–30 brpm	Respiration detection accuracy ≈ 91.2%	5	Multi-antenna setup; supervised learning; controlled sleep experiments
WiRM [36]	CSI magnitude + phase; ACF + BNR combining; AMTC spectrogram tracking; FIF-based waveform extraction	Intel 5300-class Wi-Fi NICs; 2 Tx × 2 Rx MIMO; 114 subcarriers; 9.9 Hz	∼2–5 m	8–50 brpm	>90% estimates within ±3 brpm	20	Multi-antenna setup; computationally intensive; controlled sleep experiments
Ge and Ho [37]	CSI magnitude + phase; envelope-based preprocessing; BNR-based subcarrier selection; autocorrelation peak detection	Huawei WS7002 router (Tx) + Raspberry Pi 4B (Rx, Nexmon CSI), 5.24 GHz, 80 MHz	0.7 m (Tx–Rx); subject ∼0.5 m from LOS	15–25 brpm	MAE = 0.21 brpm (magnitude), 0.30 brpm (phase)	1 (18)	Single subject; fixed-rate breathing; short-duration controlled experiments

**Table 2:** The participant demographics for the experimental study.

Item	Value
Number of participants	15
Recording duration per participant (mins)	20
Age (years)	24.1 (9.6)
Gender (males)	12 (80%)
Height (cm)	175 (±10.5)
Weight (kg)	73.3 (±12.9)
Body mass index (BMI)	23.9 (±2.6)

## Data Availability

The data presented in this study are available on request from the corresponding authors due to privacy reasons.

## Abbreviations

The following abbreviations are used in this manuscript:

ACF	Auto-correlation function
AMTC	Adaptive multi-trace carving
AoA	Angle of arrival
AP	Access point
brpm	Breaths per minute
BMI	Body Mass Index
BNR	Breathing-to-noise ratio
CSI	Channel state information
EM	Electromagnetic
FDA	Food and Drug Administration
FFT	Fast Fourier transform
FIF	Fast iterative filtering
FPC	Filtered principal component
HR	Heart rate
IIR	Infinite impulse response
LOS	Line-of-sight
MAD	Mean absolute deviation
MAE	Mean Absolute Error
MIMO	Multi-input multi-output
NIC	Network interface card
NLOS	Non-line of sight
OFDM	Orthogonal frequency-division multiplexing
PC	Principal component
PCA	Principal component analysis
RMSE	Root mean squared error
RF	Radio frequency
RR	Respiratory rate
RSSI	Received signal strength indicator
Rx	Receiver
SNR	Signal-to-noise ratio
ToF	Time of flight
Tx	Transmitter
WLAN	Wireless local area network
